# Multiparameter MRI assessment of metformin and exercise effects on skeletal muscle in prediabetes: a randomized controlled trial

**DOI:** 10.1186/s41747-025-00658-y

**Published:** 2025-12-22

**Authors:** Fuyao Yu, Yanbin Dong, Nan Ye, Chuan Xing, Xiaohong Lyu, Yong Chen, Xianghong Yang, Shinong Pan

**Affiliations:** 1 Department of Radiology, Shengjing Hospital of China Medical University, Shenyang, China; 2 Georgia Prevention Institute, Department of Medicine, Medical College of Georgia, Augusta, GA USA; 3 Department of Endocrinology, The People’s Hospital of Liaoning Province, Shenyang, China; 4 Department of Radiology, The First Affiliated Hospital of Jinzhou Medical University, Jinzhou, China; 5 Department of Radiology, The Affiliated Hospital of Hangzhou Normal University, Hangzhou, China; 6 Department of Pathology, Shengjing Hospital of China Medical University, Shenyang, China

**Keywords:** Exercise, Metformin, Multiparametric magnetic resonance imaging, Muscle, Prediabetic state

## Abstract

**Background:**

The metabolic effects of combined metformin and aerobic exercise on skeletal muscle in prediabetes remain unclear. We evaluated individual and combined effects on skeletal muscle metabolism using multiparameter magnetic resonance imaging (MRI) and assessed prediabetes remission.

**Materials and methods:**

In this 12-week randomized controlled trial, forty-two prediabetic adults aged 48.4 ± 13.1 years mean ± standard deviation) were randomized into control (*n* = 10), metformin (*n* = 10), exercise (*n* = 11), and combined therapy (*n* = 11) groups. Multiparameter MRI measured T2, apparent diffusion coefficient (ADC), fractional anisotropy (FA), intermuscular adipose tissue (IMAT), intramyocellular lipids, visceral adipose tissue (VAT), and muscle cross-sectional areas (MSCAs). Blood biomarkers were assessed by standard protocols. Analyses included ANOVA, Fisher's exact test, and correlation analysis.

**Results:**

Normoglycemia occurred in 90% of the combined group, 80% of the exercise, 20% of the metformin, and 10% of the controls. Exercise alone significantly decreased IMAT%, T2, and ADC, and increased FA, whereas metformin alone had no significant effects. Combination therapy did not further improve blood biomarkers or change MRI parameters *versus* exercise, but uniquely reduced VAT and increased MSCAs. IMAT% reduction was borderline greater in subjects with high baseline IMAT after combination therapy (*p* = 0.051). MRI parameters, particularly IMAT%, T2, and FA, correlated with fasting glucose, incremental area under the curve of glucose and insulin, and hemoglobin A1c.

**Conclusion:**

Aerobic exercise favorably altered skeletal muscle tissue characteristics and metabolic markers in prediabetes. Metformin did not attenuate these effects and may enhance IMAT reduction in subjects with high baseline IMAT.

**Relevance statement:**

Multiparameter MRI provides a sensitive, noninvasive means to quantify skeletal muscle composition and function in prediabetes, enabling precise assessment of the metabolic effects of combined lifestyle and pharmacological interventions.

**Trial registration:**

Chinese Clinical Trial Registry (ChiCTR2300072162), retrospectively registered on June 5, 2023.

**Key Points:**

- Aerobic exercise improves muscle composition and glucose metabolism in prediabetes.
- Metformin adds benefits for reducing muscle fat in high-fat individuals.
- Multiparameter MRI enables noninvasive monitoring of skeletal muscle metabolic changes.

**Graphical Abstract:**

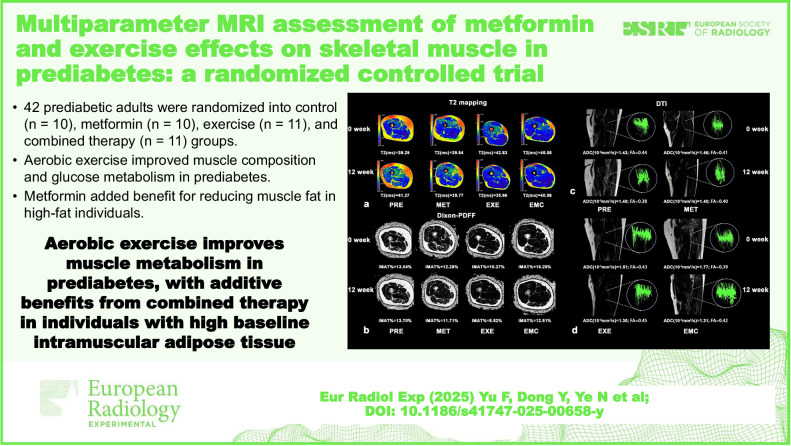

## Introduction

Prediabetes, characterized by mild glycemic dysregulation, is a major risk factor for metabolic disorders, especially type 2 diabetes mellitus (T2DM) [1]. By 2030, it is expected to affect over 470 million people worldwide [2]. The American Diabetes Association recommends that individuals with prediabetes focus on weight loss, increasing physical activity, and considering metformin therapy [3].

Beyond glycemic control and insulin resistance (IR) reduction, aerobic exercise offers numerous advantages, including enhanced aerobic capacity, increased muscular strength, and improved body composition [4]. Aerobic exercise can also reduce intermuscular adipose tissue (IMAT), which is marbled within skeletal muscle (SM) and directly related to IR. It also increases lipolysis and accelerates free fatty-acid metabolism in whole-body adipose tissues [5–8].

Metformin is commonly used to treat prediabetes and can lower both fasting and postprandial glucose levels [9]. While earlier studies attributed metformin’s glucose-lowering effects to its ability to inhibit hepatic glucose output and enhance peripheral glucose metabolism, emerging evidence suggests that the intestine also plays a significant role in mediating these effects [10].

Both moderate aerobic exercise and metformin have been shown to increase SM mass and delay the progression to T2DM in individuals with prediabetes [11, 12]. However, evidence on their combined effects remains inconsistent. Some studies suggest that metformin may attenuate the insulin-sensitizing effects of exercise, whereas aerobic exercise does not appear to diminish the hypoglycemic action of metformin and may even enhance its ability to reduce peak hyperglycemia [13–15]. Further studies have indicated that the interaction between aerobic exercise and metformin occurs primarily within SMs [16, 17]. Therefore, further clinical studies are needed to assess the individual and combined metabolic effects of metformin and aerobic exercise on SMs.

As SM biopsy is an invasive procedure, we used multiparameter magnetic resonance imaging (MRI) to assess SM composition and microstructural properties, including magnetic resonance spectroscopy (MRS), Dixon, T2 mapping, and diffusion-tensor imaging (DTI) sequences. ^1^H-MRS of SM evaluates intramyocellular lipids (IMCLs) composition [18]. Concurrently, the Dixon sequence has emerged as the preferred method for quantifying IMAT infiltration [19]. Additionally, T2 values derived from T2 mapping are quantitative imaging biomarkers of the activity of SM tissue diseases [20]. Diffusion tensor imaging (DTI) allows quantifying muscle fiber structure [21], and the apparent diffusion coefficient (ADC) and fractional anisotropy (FA) values obtained are closely associated with inflammatory myopathy [22].

We conducted a randomized controlled trial to determine the interaction between metformin and moderate aerobic exercise on SMs in patients with prediabetes. We used multiparameter MRI parameters, including IMAT, IMCL, T2, ADC, FA, muscle cross-sectional areas (MSCAs), and visceral adipose tissue (VAT), to assess SM metabolism, and evaluated glucose homeostasis and insulin sensitivity through metabolic indicators. We hypothesized that the combination of exercise and metformin would be more effective than either intervention alone.

## Materials and methods

### Experimental design

**Fig. 1:**
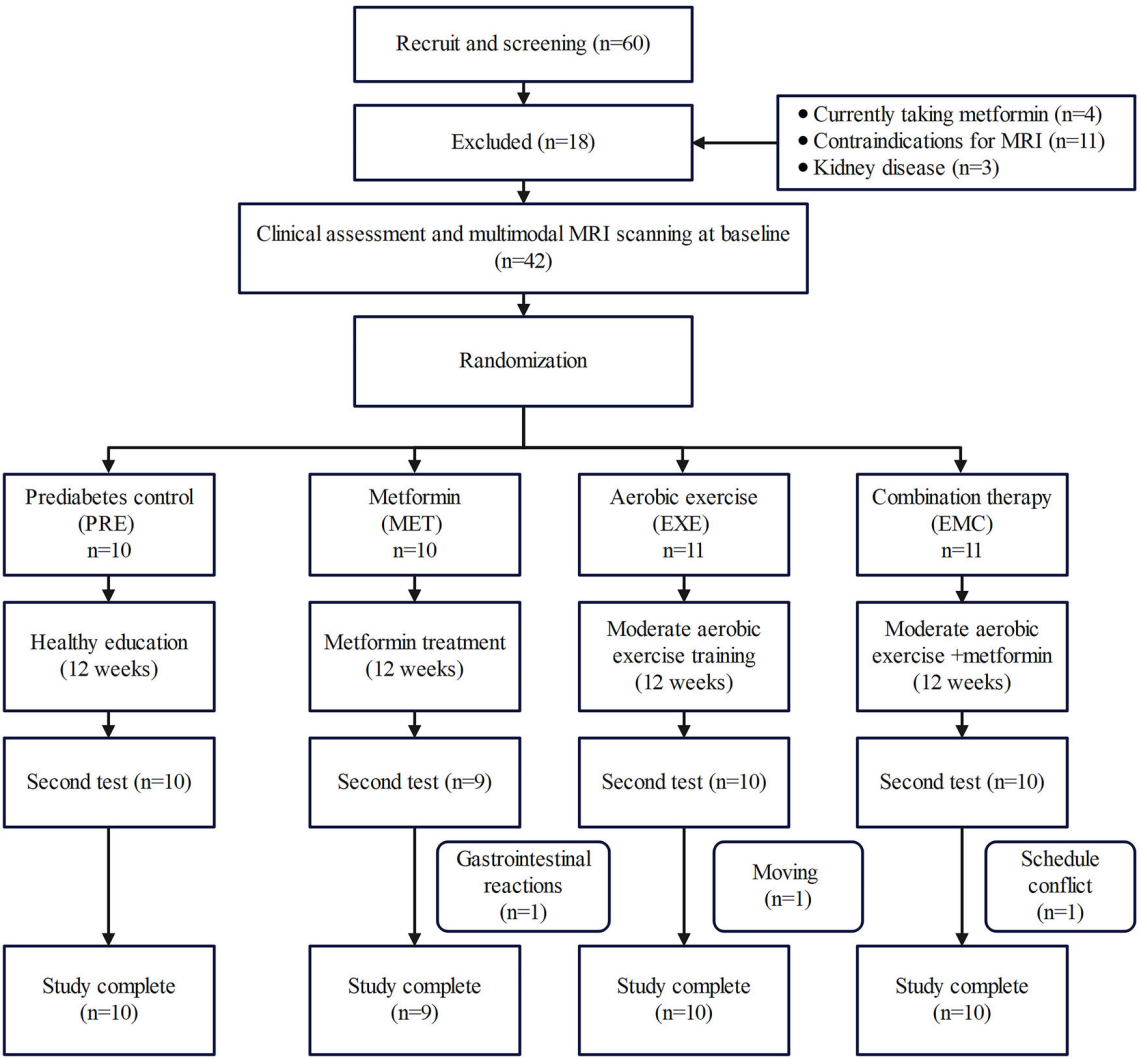
Experimental design

This randomized, controlled, open-label study involved four distinct groups. The study commenced on September 20, 2020, and concluded on August 22, 2022. The trial protocol was approved by the Institutional Ethics Committee of Shengjing Hospital of China Medical University (approval no. 2020PS596K) and registered with the Chinese Clinical Trial Registry (Registration No. ChiCTR2300072162). Our trial consisted of a baseline testing period followed by 12 weeks of active intervention. The aim was to compare the effects of the interventions at the end of the treatment period with those before the treatment. The design, conduct, and reporting of our trial adhered to the Consolidated Standards of Reporting Trials–CONSORT guidelines. Figure 1 illustrates the experimental design.

### Participants

Forty-two adults with prediabetes, including impaired fasting glucose and/or impaired glucose tolerance, were recruited for this study. The enrollment criteria were as follows: (1) clinical diagnosis of prediabetes (fasting blood glucose [FBG] range: 6.1–6.9 mmol/L and/or 2-h postprandial glucose [oral glucose tolerance test] range: 7.8–11.0 mmol/L); (2) no contraindications to MRI scanning; and (3) age of 25–65 years. The exclusion criteria were as follows: (1) clinically confirmed T2DM; (2) acute liver or kidney disease; (3) acute infection, fever, trauma, or other endocrine diseases that may affect blood glucose; (4) acute cerebrovascular disease, coronary artery disease, chronic cardiac insufficiency, or hyperuricemia; (5) strenuous exercise within the preceding 4 weeks; and (6) taking metformin within the preceding three months.

Participants were recruited through public advertisements and from the endocrinology outpatient clinics and the health examination center in Shengjing Hospital of China Medical University. All participants provided written informed consent before participating in the study. Participants did not receive any monetary or other rewards for participating in this trial, but their travel expenses from home to the clinic were reimbursed.

### Randomization

We utilized centralized randomization to guarantee an impartial allocation of groups. The allocation sequence was produced by an independent statistician using the *randomizeR* package in the R software (version 4.0.0). An independent statistician generated the random allocation sequence. Study coordinators enrolled participants and assigned them to interventions. The group assignment for patients was only disclosed after they met the criteria for the study and provided their consent to participate. Briefly, the participants were randomized into control (PRE; *n* = 10), metformin (MET; *n* = 10), exercise-only (EXE; *n* = 11), and combined metformin and exercise group (EMC; *n* = 11) groups.

### Interventions

All participants received general advice on diet based on national recommendations [23]. Moreover, all included subjects received written instructions to abstain from alcohol for ≥ 48 h prior to each fasting blood draw and MRI examination and not to increase their habitual intake during the 12‑week intervention. Moderate-intensity aerobic exercise (MIAE) was defined as maintaining a heart rate at 50% of the age-predicted maximum (197 − 0.8 × age for women; 204 − 0.9 × age for men) [24]. Participants in the EXE and EMC groups performed jogging 5 days per week for 30 min per session. The intensity of the exercise in all groups was objectively monitored using Huawei Band 4 (Huawei Technologies Co., Ltd.). The device ensured real-time tracking of the participants’ heart rates to maintain the desired exercise intensity. All data were automatically uploaded and stored on the Huawei Health app platform (version 9.0.6, Huawei Technologies Co., Ltd.), allowing for a comprehensive analysis of the participants’ adherence to the prescribed exercise intensity and duration. The last exercise session was scheduled to occur at least 48 h prior to any subsequent measures to mitigate the potential impact of acute exercise on the outcomes.

The MET and EMC groups were given metformin hydrochloride extended-release tablets (H20023370: Merck Pharmaceutical Manufacturing [Jiangsu] Co., Ltd.) at a dose of 1,500 mg per day (750 mg at 8 a.m. and 750 mg at 8 p.m.) [25].

### Blood testing

All participants were required to fast from 10:00 p.m. overnight. Blood samples were collected at 8:00 a.m. the following morning in the hospital’s laboratory. Participants adhered to a standard diet, provided by the investigators based on national recommendations [23], for 24 h prior to blood collection. They were also instructed to abstain from both prescription and over-the-counter medications for 48 h preceding the sampling. Strenuous physical activities were prohibited for 24 h before the collection, and participants were advised to ensure adequate sleep the night before.

Blood was collected at baseline (week 0) and post-intervention (week 12). FBG, fasting blood insulin (FINS), and hemoglobin A1c (HbAlc) levels were measured. For the oral glucose tolerance test, participants consumed 75 g of anhydrous glucose in 200 mL of water within 3 min, and venous blood was drawn at 1 and 2 h to assess 1-h postprandial blood glucose (1hPG), 2-h postprandial blood glucose (2hPG), 1-h-insulin, and 2-h insulin levels. Blood glucose was measured using the glucose oxidase method [26]. Serum insulin was analyzed using an electro-chemiluminescence immunoassay (Cobas e411; Roche Diagnostics) after samples for serum insulin analysis were centrifuged 30 min post-collection. HbA1c was measured by HPLC (Tosoh G8; Tosoh Corporation). The homeostasis model assessment (HOMA) method was used to quantify IR, using: HOMA–IR = FBG × FINS/22.5.

### Multiparameter MRI

Following blood sampling, participants immediately underwent multi‑parameter MRI on an Ingenia 3.0-T system (Philips Healthcare). All examinations were performed in the supine, head‑first position by a radiographer with over 3 years of experience. The thigh acquisition covered the region from the anterior superior iliac spine to just above the knee joint. Sagittal, coronal, and axial T1-weighted and T2-weighted scans were acquired both for anatomical reference and for quantifying mid-thigh MSCAs. Quantitative sequences comprised axial T2 mapping, DTI, and a multi‑echo (six‑echo) gradient‑echo Dixon sequence, which was used to derive proton density fat fraction (PDFF) maps for IMAT quantification. In particular, DTI was performed using a multislice spin-echo single-shot echo-planar imaging sequence covering the same anatomical region as the Dixon acquisition. The protocol included 15 noncolinear diffusion encoding directions plus one baseline (*b* = 0) image, with two b-values (0 and 500 s/mm²). The number of signal averages was 2. Signal-to-noise ratio was estimated on the *b* = 0 images using a previously validated difference method. With a region of interest (ROI) size of approximately 120 mm² (≈53 voxels) and assuming a voxel-wise signal-to-noise ratio of ≈10, the resulting ROI-averaged signal-to-noise ratio was estimated at ~103, exceeding the commonly accepted signal-to-noise ratio threshold (~60) for reliable FA and ADC quantification [27]. Based on our recent *in vivo* preclinical study [28], the vastus lateralis was interrogated with single‑voxel ¹H‑MRS, acquired using a point-resolved spectroscopy with chemical shift selective imaging water suppression; spectra were quantified with the vendor‑embedded LCModel algorithm, with no T1 or T2 relaxation correction applied during metabolite quantification. Abdominal axial T2-weighted images were acquired at the L3–L4 intervertebral level for VAT assessment. Detailed scanning parameters are provided in Supplemental Table S1.

MRI measurements included: (1) the percentage of IMAT in the SMs (IMAT%) and MSCA in the mid-thigh; (2) T2, FA, ADC values, and intramyocellular lipid to creatinine ratio (IMCL/Cr) in the vastus lateralis; and (3) VAT area, obtained from abdominal scans at the L3–L4 vertebral level [29].

### Procedures and outcomes

#### Outcomes

The primary efficacy outcome is the remission rate of prediabetes at the end of treatment, which is calculated as the percentage of individuals in each group with FBG < 6.1 mmol/L and 2 h PG < 7.8 mmol/L. Secondary outcomes included IMAT%, MSCAs, T2, ADC, FA, and IMCL/Cr values of SM, VAT area at the L3–L4 vertebral level, HbA1c, FBG, FINS, body mass index (BMI), and plasma glucose and serum insulin levels during the 2-h oral glucose tolerance test. Except for sex and the proportions of IFG and IGT, all study variables were continuous. Analyses of secondary endpoints were considered exploratory in nature.

#### Clinic visits

All participants were interviewed or contacted by phone once per week. Participants in the EXE and EMC groups were asked to record their daily exercise through a micro-application (WeChat version 8.0.25: Tencent Technology). Patients in the MET and EMC groups were asked to record their daily use of medication through another micro-application.

### Quantitative MRI analysis

T2-mapping, ^1^H-MRS, DTI, and Dixon datasets were processed on Philips Research Imaging Development Environment version 4.1 (Philips N.V.). In particular, T2 values were derived using pixel-wise mono-exponential fitting of multi-echo sequences, with the initial echo excluded to minimize the influence of stimulated echoes, and without the application of fat suppression, as previously described [30]. During ROI placement, non-muscle regions such as subcutaneous fat and fascia were carefully excluded to ensure that only the muscles were analyzed. Under these acquisition conditions, T2 values functioned as global surrogate markers of SM composition. DTI datasets were processed using the FiberTrak module (Philips Research Imaging Development Environment version 4.1). Color-coded FA and ADC maps were co-registered with Dixon water-fat images to ensure anatomical reference. Fiber tracking was performed using a single-ROI line-propagation technique. According to previous literature [31], tracking was initiated from seed ROIs using the following thresholds: FA > 0.12 and direction change ≤ 6.75°. After fiber tracking, mean FA and ADC values were automatically generated by the software. These quantitative parameters were extracted from ROIs measuring approximately 120 mm² to reduce noise-related variability and partial volume effects. Dixon datasets were reconstructed into water-only, fat-only, and PDFF maps using the vendor’s multi-echo water–fat separation algorithm. IMAT% was quantified on mid-thigh PDFF maps by manually delineating the deep fascial boundary on each slice using the Philips workstation. Subcutaneous fat was excluded, and voxels within the intrafascial compartment showing signal characteristics consistent with adipose tissue were labeled. IMAT% was calculated as the ratio of intrafascial fat signal to the total intrafascial signal. MSCA and VAT measurements were performed using standardized anatomical landmarks and manual segmentation techniques. Mid-thigh MSCAs were measured on axial T1-weighted images at the mid-thigh level, defined as the midpoint between the anterior superior iliac spine and the superior patellar border. Manual segmentation was conducted using Mimics Research version 21.0 (Materialise NV), aided by thresholding tools to delineate the intrafascial muscle area. VAT was assessed on axial T2-weighted images acquired at the L3–L4 intervertebral level. The visceral fat compartment was manually outlined within the abdominal cavity using the same software, with careful exclusion of subcutaneous and paraspinal fat. The resulting VAT area was automatically calculated by the program.

Three independent readers with over 3 years of experience in musculoskeletal MRI performed all quantitative image analyses. To assess intrareader agreement for multiparameter MRI, all readers repeated the analyses within 2 weeks, to avoid recall bias. The readers were blinded to all laboratory and grouping information.

### Monitoring adverse events and assessing treatment adherence

Adverse events were meticulously recorded and their severity assessed during the visits at 6 and 12 weeks. In the metformin group (MET), we evaluated treatment adherence by quantifying the amount of medication that participants returned at the 6 and 12-week check-ups. Individuals were deemed compliant if they had consumed at least 80% of their prescribed doses. In the context of the exercise intervention, adherence was gauged based on the quantity and length of completed exercise sessions. Participants were labeled as adherent if they accomplished at least 80% of the assigned exercise sessions and total exercise volume. Severity was evaluated using the Common Terminology Criteria for Adverse Events version 5.0. Participants were instructed to immediately notify the investigators of any adverse events occurring between scheduled visits. All reported adverse events were documented and evaluated for severity by the investigators.

### Sample size determination and power analysis

The primary outcome of this study was the remission rate of prediabetes at the end of the 12-week intervention, defined as meeting both FBG < 6.1 mmol/L and 2h-PG < 7.8 mmol/L criteria. As the remission rates for 12-week interventions with metformin and/or aerobic exercise in prediabetes are not firmly established, we estimated plausible group differences based on prior studies. Specifically, we assumed remission rates of 15% in the MET [25], 35% in the aerobic EXE [32], and 65% in the EMC [33]. Under these assumptions, we conducted a power analysis for a three-group chi-square test (d*f* = 2), with a significance level of α = 0.05 and statistical power of 80%. The resulting non-centrality parameter was λ₀.₈ ≈ 9.64. Given an estimated effect size of *w*² = 0.3306, the required total sample size for the three intervention groups (MET, EXE, and EMC) was calculated to be approximately 30. The prediabetes cohort (PRE) was included only as an observational reference and therefore excluded from the formal power calculation. Adding a group of comparable size increased the planned total sample to about 40 participants, and we consequently enrolled 10–11 individuals in each of the four groups.

### Statistical analysis

GraphPad Prism v 8.0 (GraphPad Software Inc.) and SPSS v 23.0 for Windows (IBM Corp.) were used for all statistical analyses. Following normality and Chi-squared tests, data that conformed to a normal distribution were expressed as mean ± standard deviation, whereas data that did not were expressed as median (interquartile range). Intraclass correlation coefficients (ICCs) with 95% confidence intervals were calculated using two-way mixed-effects models to determine the intra- and inter-reader consistency of agreement. Reverse rates from prediabetics to normal glycemic conditions (FBG < 6.1 mmol/L, 2-h-oral glucose tolerance test < 7.8 mmol/L) were compared between groups using Fisher’s exact test. The primary outcome was analyzed using the intention-to-treat approach to ensure an unbiased assessment of treatment effects. Since intention-to-treat and per-protocol results were similar for the primary outcome, per-protocol analysis was used for secondary outcomes to explore treatment efficacy in participants who adhered to the intervention. One-way analysis of variance (ANOVA) was used to compare differences between multiple groups for data with homogeneity of variance. Two-way repeated measures ANOVA was used to examine the differences between pre-treatment and post-treatment. All ANOVAs were followed by least significant difference (LSD) post hoc tests. For data that did not meet the assumption of homogeneity of variance, nonparametric tests were employed. Partial correlation was used to analyze the association between the two data groups. All *p*-values were two-sided, with statistical significance set at *p* < 0.05.

## Results

### Follow-up

Of 42 initial participants, 39 (93%) completed the 12-week follow-up period. In the PRE group, all ten participants (100%) completed the study. In the MET group, one participant was lost due to gastrointestinal reactions, resulting in nine completions (90%). In the EXE group, one participant moved, resulting in 10 completions (91%). In the EMC group, one participant had a schedule conflict, resulting in 10 completions (91%). The study commenced on September 20, 2020, and concluded on August 22, 2022. The trial was ended as scheduled. Participants who were lost to follow-up were directly excluded from the data analysis.

### Baseline patient characteristics

Each group analysis included the number of participants (denominator) based on the original assigned groups (Table 1). No significant differences were found between groups in terms of age, sex, BMI, or metabolic and insulin markers (FBG, 1hPG, 2hPG, HbAlc, FINS, 1-h insulin, 2-h insulin, homeostasis model assessment of insulin resistance (HOMA-IR), incremental area under the curve (iAUC) for glucose and insulin). Quantitative multiparameter MRI analysis also revealed no significant variations in IMAT%, VAT, T2, ADC, FA, IMCL/Cr, and MSCAs across all participant groups (Table 2).

**Table 1:** Comparison of characteristics of study participants

	PRE	MET	EXE	EMC
Age (years)	52.3 ± 12.3	49.5 ± 15.0	43.5 ± 13.2	48.8 ± 12.1
Sex (male%)	40.0%	50.0%	54.5%	45.5%
Sex (female%)	60.0%	50.0%	45.5%	54.5%
Isolated IGT	2 (10)	1 (10)	1 (11)	2 (11)
Isolated IFG	1 (10)	1 (10)	2 (11)	1 (11)
Combined IFG and IGT	7 (10)	8 (10)	8 (11)	8 (11)
BMI (kg/m^2^)	24.3 ± 3.0	27.1 ± 5.1	26.7 ± 5.0	25.3 ± 4.3
FBG (mmol/L)	6.3 ± 0.3	6.5 ± 0.3	6.3 ± 0.5	6.5 ± 0.3
1hPG (mmol/L)	10.6 ± 1.3	10.7 ± 1.3	9.7 ± 1.3	9.8 ± 0.9
2hPG (mmol/L)	9.2 ± 1.3	9.0 ± 1.1	8.5 ± 1.1	8.5 ± 0.9
HbAlc (%)	6.0 ± 0.4	6.2 ± 0.2	6.1 ± 0.3	5.9 ± 0.4
FINS (mU/L)	13.0 (8.1, 13.8)	11.7 (5.5, 13.7)	12.3 ± 7.4	11.9 ± 5.8
1-h insulin (mU/L)	98.8 (43.7, 144.5)	74.8 ± 41.5	88.5 ± 46.9	69.2 ± 29.3
2-h insulin (mU/L)	68.9 ± 61.6	49.3 ± 26.5	56.9 ± 41.9	34.8 ± 32.1
HOMA-IR	3.6 (2.3, 3.8)	3.4 (1.6, 4.6)	3.5 ± 2.3	3.4 ± 1.7
iAUC _glucose_ (mmol/L × min)	18.4 ± 1.9	18.4 ± 1.8	17.1 ± 1.8	17.3 ± 1.3
iAUC _insulin_ (mU/L × min)	139.8 (55.2, 234.8)	123.1 ± 68.0	118.2 ± 69.7	92.6 ± 30.8

*1hPG* 1-h postprandial glucose, *2hPG* 2-h postprandial glucose, *EMC* Combined metformin and moderate aerobic exercise group, *EXE* Moderate aerobic exercise group, *FBG* Fasting blood glucose, *FINS* Fasting blood insulin, *HbA1c* Hemoglobin A1c, *HOMA-IR* Homeostasis model assessment of insulin resistance, *iAUC* Incremental area under the curve, *MET* Metformin treatment group, *PRE* Prediabetes group

**Table 2:** Comparisons of multimodal MRI parameters of study participants

	PRE	MET	EXE	EMC
IMAT%	13.00 ± 1.28	12.93 ± 4.40	11.00 ± 2.31	11.54 ± 2.57
T2 (ms)	39.27 ± 1.45	41.14 ± 1.73	40.06 ± 2.38	40.06 ± 2.31
ADC (10^-3^ mm^2^/s)	1.37 ± 0.12	1.40 ± 0.11	1.43 ± 0.17	1.50 ± 0.15
FA	0.40 ± 0.08	0.41 ± 0.05	0.41 ± 0.04	0.41 ± 0.05
MSCA (cm^2^)	116.14 ± 24.38	108.91 ± 26.60	115.70 ± 29.69	117.00 ± 28.07
IMCL/Cr	10.41 ± 2.85	15.16 ± 10.18	11.48 ± 7.31	8.25 ± 2.62
VAT (cm^2^)	122.99 ± 43.94	170.31 ± 83.22	126.54 ± 41.76	119.51 ± 48.04

*ADC* Apparent diffusion coefficient, *Cr* Creatinine, *EMC* Combined metformin and moderate aerobic exercise group, *EXE* Moderate aerobic exercise group, *FA* Fractional anisotropy, *IMAT* Intermuscular adipose tissue, *IMCL* Intramuscular lipid, *MET* Metformin treatment group, *MSCA* Muscle cross-sectional area, *PRE* Prediabetes group, *VAT* Visceral adipose tissue

### Intra- and inter-reader agreement

Intra-reader agreement was high for all multiparameter MRI (ICC > 0.80). Similarly, inter-reader reproducibility was excellent for the assessment of T2 (ICC, 0.92 or 0.85), ADC (ICC, 0.93 or 0.83), FA (ICC, 0.93 or 0.94), VAT (measured in cm^2^; ICC, 0.93 or 0.89), IMAT% (ICC, 0.96 or 0.84) and MSCAs (0.96 or 0.92) (Supplementary Table S2).

### Primary outcome

By the end of the study, nine participants (90%) in the EMC group, eight (80%) in the EXE group, two (20%) in the MET group, and one (10%) in the PRE group achieved normoglycemia from a prediabetic state (Table 3). In the intention-to-treat analysis, the EXE and EMC groups had significantly higher reversal rates than PRE (*p* = 0.0055 and *p* = 0.0011, respectively), with relative risks (RR) of 8.0 (95% confidence interval: 1.21, 52.69) and 9.0 (1.39, 58.45), and absolute risk differences (ARD) of +70% (39.0%, 101.0%) and +80% (53.7%, 106.3%), respectively. By contrast, the MET group did not differ significantly from PRE (RR = 2.0, *p* = 1.0000, ARD = +10%). Per-protocol analysis yielded similar findings, with EXE (*p* = 0.0011) and EMC (*p* = 0.0001) both showing significant improvements over PRE, while MET remained non-significant (*p* = 0.5820). No significant difference was observed between EXE and EMC in either analysis.

**Table 3:** Clinical reverse rates of prediabetes

Group	Reverse rate (ITT)	Reverse rate (PP)	Absolute risk difference (95% CI)	Relative risk (95% CI)	Number needed to treat (NNT)	*p*-value (ITT)	*p*-value (PP)
PRE	10.0% (1/10)^c,d^	10.0% (1/10)^c,d^	Reference	Reference	Reference	Reference	Reference
MET	20.0% (2/10)^c,d^	22.2% (2/9)^c,d^	+10% (–21.0%, +41%)	2.0 (0.21, 18.69)	10.00	1.0000	0.5820
EXE	80.0% (8/10)^a,b^	88.9% (8/9)^a,b^	+70% (+39.0%, +101%)	8.0 (1.21, 52.69)	1.43	0.0055	0.0011
EMC	90.0% (9/10)^a,b^	100% (9/9)^a,b^	+80% (+53.7%, +106.3%)	9.0 (1.39, 58.45)	1.25	0.0011	0.0001

*ARD* Absolute risk difference, *EMC* Combined metformin and moderate aerobic exercise group, *EXE* Moderate aerobic exercise group, *ITT* Intention-to-treat, *MET* Metformin treatment group, *NNT* Number needed to treat, *PP* Per-protocol, *PRE* Prediabetes group, *Ref* Reference group (PRE) for *p*-value comparisons, *RR* Relative risk

The criteria for clinical reverse of prediabetes were FBG < 6.1 mmol/L and 2 h postprandial blood glucose < 7.8 mmol/L

^a^ *p* < 0.05, significantly different from the PRE group

^b^ *p* < 0.05, significantly different from the MET group

^c^ *p* < 0.05, significantly different from the EXE group

^d^ *p* < 0.05, significantly different from the EMC group

### Secondary outcomes: effects on BMI, glucose control, and insulin sensitivity

After 12 weeks, both the EXE (*p* = 0.004) and EMC (*p* < 0.001) groups showed significant decreases in BMI compared to pre-treatment values, while only the EMC group had significantly lower BMI than the PRE (*p* = 0.049) and MET groups (*p* = 0.020) (Table 4).

**Table 4:** Inter- and intra-group comparisons of blood glucose homeostasis indicators and insulin sensitivity among the groups before and after treatment

	PRE	MET	EXE	EMC
BMI (kg/m^2^)	25.8 ± 2.8^d^	26.5 ± 4.2^d^	24.7 ± 3.9^&^	22.7 ± 2.6^a,b,&^
FBG (mmol/L)	7.2 ± 1.9^b,c,d,&^	5.9 ± 0.7^a^	5.3 ± 0.9^a^	5.3 ± 0.7^a,&^
1hPG (mmol/L)	11.8 ± 2.0^c,d^	12.2 ± 4.1^c,d^	7.8 ± 2.0^a,b^	6.5 ± 1.1^a,b,&^
2hPG (mmol/L)	11.4 ± 4.0^c,d^	11.1 ± 4.9^c,d^	6.0 ± 1.4^a,b^	5.0 ± 1.3^a,b,&^
HbAlc (%)	6.5 ± 1.3^d^	6.5 ± 1.5^d^	5.7 ± 0.4	5.6 ± 0.4^a,b^
FINS (mU/L)	13.8 (8.4, 16.0)^a^	9.1(7.5, 10.35)	8.8 ± 4.9^&^	8.3 ± 2.2^d,&^
1-h insulin (mU/L)	120.9 (52.3, 196.7)	74.7 ± 35.9	77.3 ± 41.6	55.8 ± 30.8
2-h insulin (mU/L)	73.4 ± 60.5	42.3 ± 18.6	42.1 ± 28.8	28.5 ± 25.8
HOMA-IR	4.3 ± 2.5^b,c,d^	2.4 ± 0.8^a^	2.2 ± 1.2^a,&^	1.9 ± 0.5^a,&^
iAUC _glucose_ (mmol/L × min)	21.1 ± 3.5 ^c,d^	20.8 ± 6.6 ^c,d^	13.5 ± 2.7^a,b,&^	11.6 ± 1.8^a,b,&^
iAUC _insulin_ (mU/L × min)	164.5 (77.9, 292.5)	100.4 ± 41.4	102.8 ± 55.8	85.1 ± 29.74

*1hPG* 1-h postprandial glucose, *2hPG* 2-h postprandial glucose, *EMC* Combined metformin and moderate aerobic exercise group, *EXE* Moderate aerobic exercise group, *FBG* Fasting blood glucose, *FINS* Fasting blood insulin, *HbA1c* Hemoglobin A1c, *HOMA-IR* Homeostasis model assessment of insulin resistance, *iAUC* Incremental area under the curve, *MET* Metformin treatment group, *PRE* Prediabetes group

^&^ *p* < 0.05 indicates intra-group significant differences before and after treatment

^a^ *p* < 0.05, significantly different from the PRE group

^b^ *p* < 0.05, significantly different from the MET group

^c^ *p* < 0.05, significantly different from the EXE group

^d^ *p* < 0.05, significantly different from the EMC group

Regarding glycemic control, FBG levels were significantly lower in all treatment groups compared to the PRE group (*versus* MET: *p* = 0.024; *versus* EXE: *p* = 0.001; *versus* EMC: *p* < 0.001). However, only the EMC group showed a significant reduction in FBG from baseline (*p* < 0.001). The 1hPG, 2hPG, and iAUC-glucose levels significantly decreased in the EXE and EMC groups compared to both the PRE and MET groups (*p* < 0.001). In intra-group comparisons, only the EMC group exhibited significant reductions in 1hPG (*p* = 0.001) and 2hPG (*p* = 0.001) from baseline, while both EXE (*p* = 0.042) and EMC (*p* = 0.001) groups showed significant decreases in iAUC-glucose. Additionally, HbA1c levels were significantly lower in the EMC group compared to the PRE (*p* = 0.037) and MET (*p* = 0.036) groups.

For insulin sensitivity, HOMA-IR levels significantly decreased in all treatment groups (MET: *p* = 0.010; EXE: *p* = 0.003; EMC: *p* = 0.001). However, only the EMC group showed a significant reduction in FINS compared to the PRE group (*p* = 0.027). Moreover, both the EXE and EMC groups demonstrated significant decreases in FINS (EXE: *p* = 0.043; EMC: *p* = 0.008) and HOMA-IR (EXE: *p* = 0.019; EMC: *p* = 0.002) relative to baseline.

### Multiparameter MRI outcomes following intervention

All MRI parameters used for inter- and intra-group comparisons were absolute values measured at the end of the 12-week intervention, following the completion of all treatments.

**Fig. 2:**
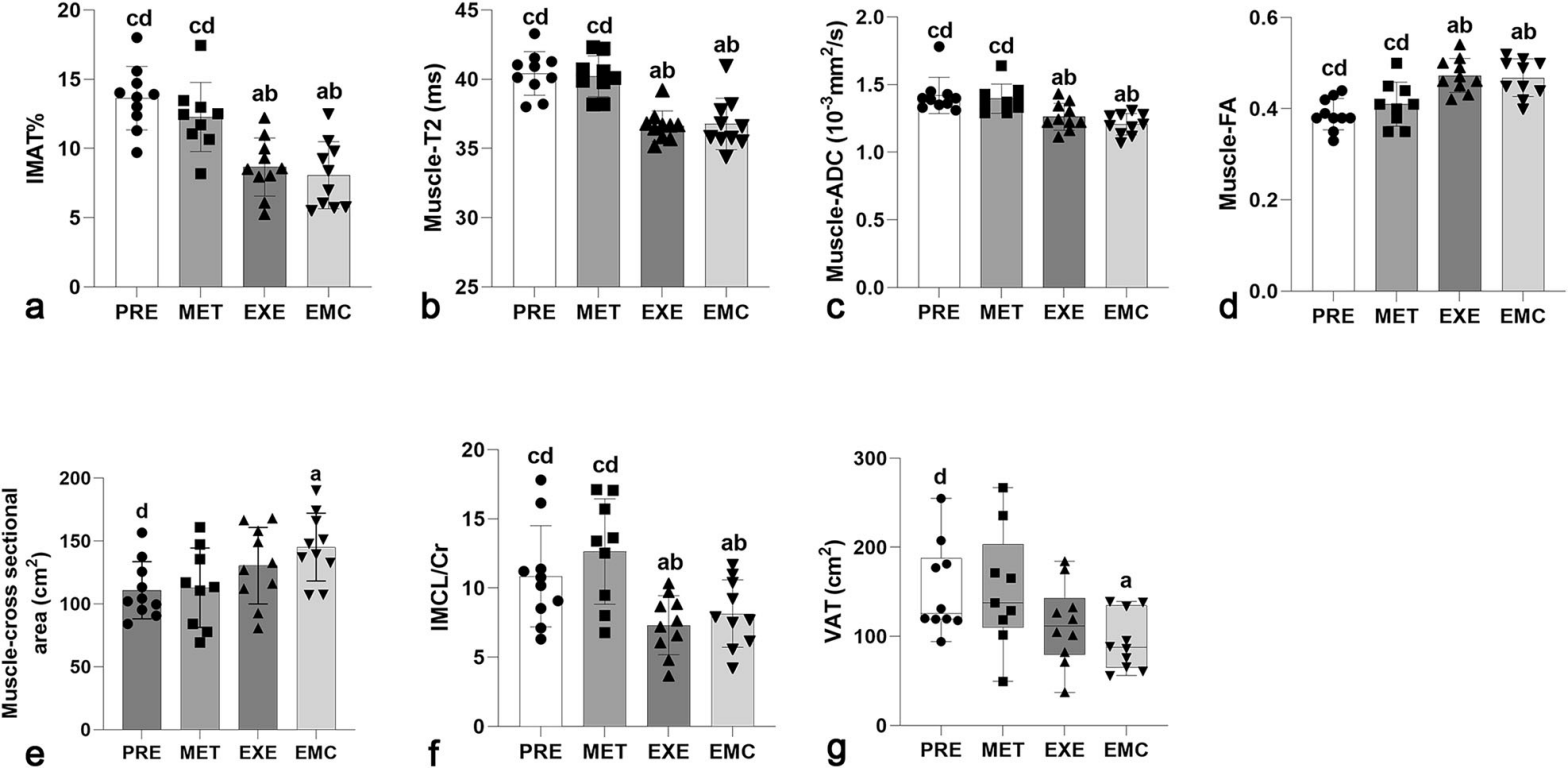
Post-intervention inter-group comparison of SM multiparameter MRI parameters. ADC, Apparent diffusion coefficient; Cr, Creatinine; EMC, Combined metformin and moderate aerobic exercise group; EXE, Moderate aerobic exercise group; FA, Fractional anisotropy; IMAT, Intermuscular adipose tissue; IMCL, Intramuscular lipid; MET, Metformin treatment group; PRE, Prediabetes group; VAT, Visceral adipose tissue. Statistical plots of: IMAT% (**a**); muscle-T2 (**b**); muscle-ADC (**c**); muscle-FA (**d**); muscle-cross-sectional areas (**e**); IMCL/Cr (**f**); and VAT (cm^2^) (**g**). ^a^ *p* < 0.05, significantly different from the PRE group; ^b^ *p* < 0.05, significantly different from the MET group; ^c^ *p* < 0.05, significantly different from the EXE group; AND ^d^ *p* < 0.05, significantly different from the EMC group. Data are expressed as mean ± standard deviation. All values represent absolute measurements acquired at the end of the 12-week intervention, after completion and cessation of metformin and/or exercise treatment

For inter-group comparisons (Fig. 2), the EXE and EMC groups showed significantly lower T2 (*versus* PRE: *p* < 0.001 and < 0.001; *versus* MET: *p* < 0.001 and < 0.001), ADC (*versus* PRE: *p* = 0.001 and < 0.001; *versus* MET: *p* = 0.047 and 0.003), and IMAT% (*versus* PRE: *p* < 0.001 and < 0.001; *versus* MET: *p* = 0.001 and 0.002) values, and significantly higher FA values (*versus* PRE: *p* < 0.001 and 0.001; *versus* MET: *p* = 0.001 and 0.020), respectively. A significant reduction in VAT was observed only in the EMC group relative to the PRE group (*p* = 0.043). IMCL/Cr values were significantly lower in the EXE and EMC groups compared to the MET (*versus* EXE: *p* = 0.003; *versus* EMC: *p* = 0.016) and PRE (*versus* EXE: *p* = 0.040; *versus* EMC: *p* = 0.049) groups. However, no significant differences in any muscle MRI parameters were found between the EXE and EMC groups.

**Fig. 3:**
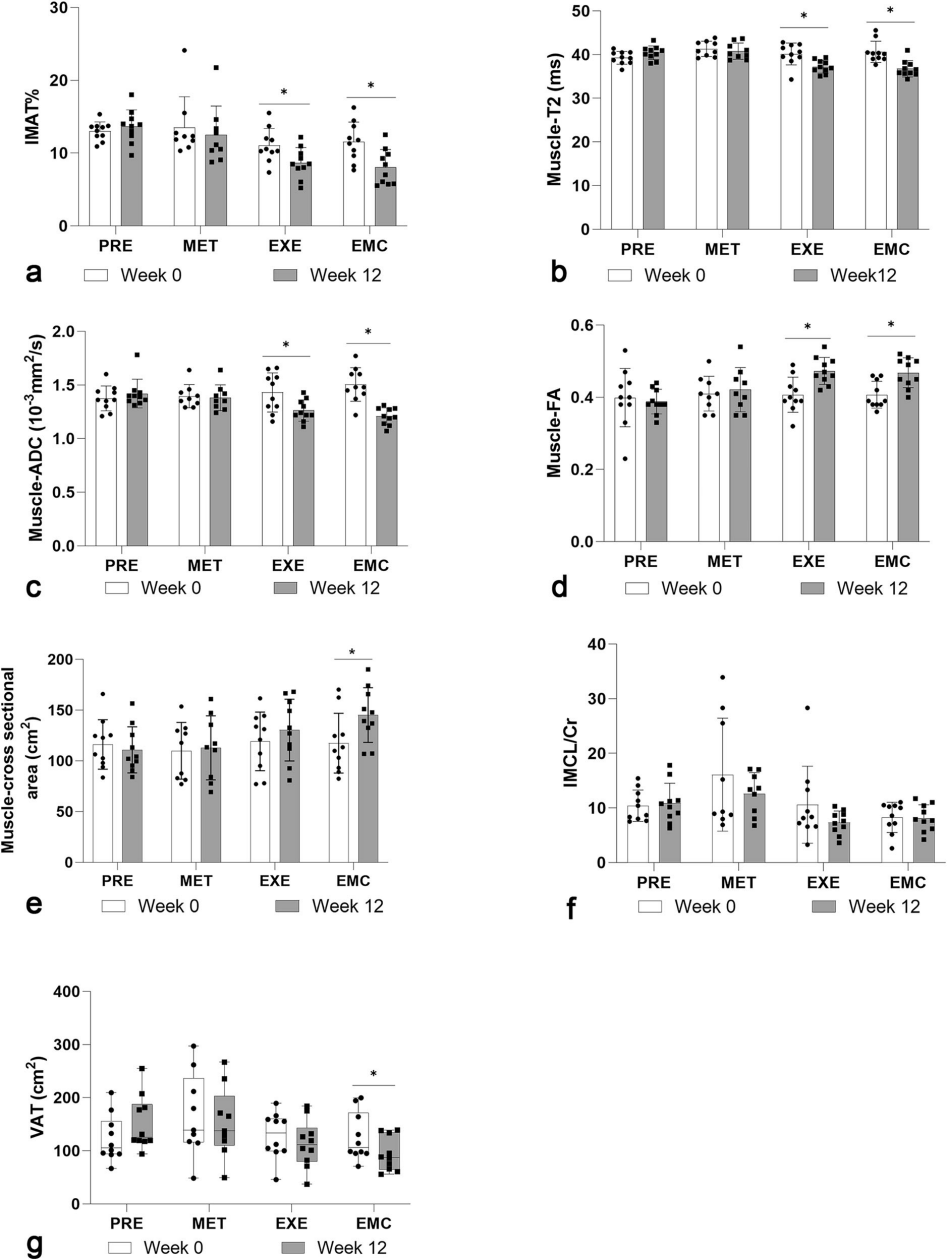
Intra-group comparison of changes in SM multiparameter MRI parameters. ADC, Apparent diffusion coefficient; Cr, Creatinine; EMC, Combined metformin and moderate aerobic exercise group; EXE, Moderate aerobic exercise group; FA, Fractional anisotropy; IMAT, Intermuscular adipose tissue; IMCL, Intramuscular lipid; MET, Metformin treatment group; PRE, Prediabetes group; VAT, Visceral adipose tissue. Statistical plots of intra-group comparison of: IMAT% (**a**); muscle-T2 (**b**); muscle-ADC (**c**); muscle-FA (**d**); MSCAs (**e**); IMCL/Cr (**f**); and VAT (cm^2^) (**g**). * *p* < 0.05, significantly different before and after treatment. Data are expressed as mean ± standard deviation

For intra-group comparisons (Fig. 3), both the EXE and EMC groups showed significant decreases in T2 (EXE: *p* = 0.002; EMC: *p* < 0.001), ADC (EXE: *p* = 0.001; EMC: *p* < 0.001), IMAT% (EXE: *p* = 0.001; EMC: *p* < 0.001), and VAT (EXE: *p* = 0.049; EMC: *p* = 0.045), as well as significant increases in FA (EXE: *p* = 0.001; EMC: *p* = 0.002) after the intervention. Additionally, only the EMC group exhibited a significant increase in MSCAs (*p* < 0.001) compared to baseline. No significant changes in IMCL/Cr were observed within any group.

**Fig. 4:**
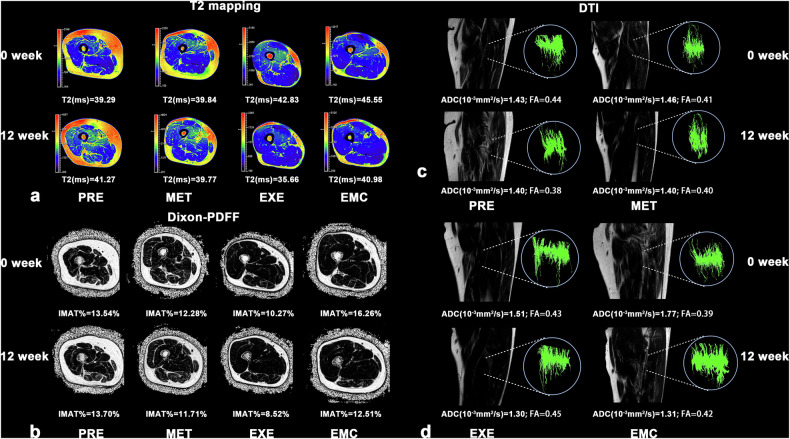
Main characteristics of changes in multiparameter MRI of SM across groups. ADC, Apparent diffusion coefficient; DTI, Diffusion-tensor imaging; EMC, Combined metformin and moderate aerobic exercise group; EXE, Moderate aerobic exercise group; FA, Fractional anisotropy; IMAT, Intermuscular adipose tissue; MET, Metformin treatment group; PDFF, Proton density fat fraction; PRE, Prediabetes group

Figure 4 presents representative multiparameter MRI scans acquired before and after treatment in each group. Notably, representative T2 mapping and Dixon-PDFF images (Fig. 4a, b) showed reduced T2 values and IMAT% at 12-week follow-up in the EXE and EMC groups. Meanwhile, muscle fiber tractography (Fig. 4c, d) suggested a slight reduction in fiber density in the PRE group, whereas fiber continuity and organization appeared preserved or slightly improved following exercise interventions.

### Post-treatment IMAT% change in all participants and stratified subgroups

**Fig. 5:**
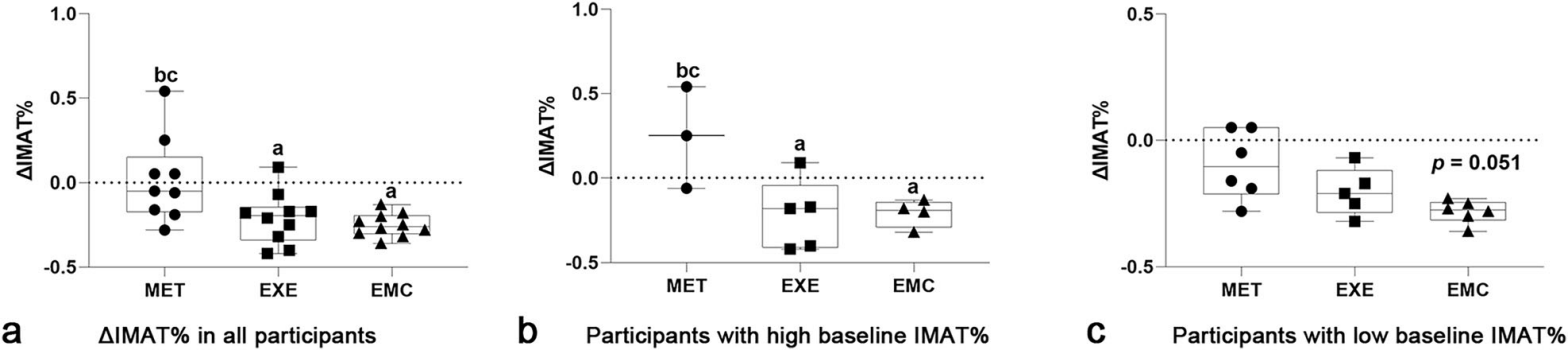
Group differences in post-treatment change rates in IMAT%. EMC, Combined metformin and moderate aerobic exercise group; EXE, Moderate aerobic exercise group; IMAT, Intermuscular adipose tissue; MET, Metformin treatment group; PRE, Prediabetes group. ^a^ *p* < 0.05, significantly different from the MET group; ^b^ *p* < 0.05, significantly different from the EXE group; ^c^ *p* < 0.05, significantly different from the EMC group; *p* = 0.051, refers to the difference between MET and EMC in participants with high baseline IMAT%; ΔIMAT% represents the absolute change in IMAT percentage from baseline to week 12 (week 12 – week 0)

The post-treatment change in IMAT% (ΔIMAT%) showed a significantly greater reduction in the EXE (*p* = 0.020) and EMC (*p* = 0.006) groups compared to the MET group (Fig. 5a). We further used the median IMAT% (11.78%) to stratify the participants into two treatment groups: low (< 11.78%) and high IMAT% (≥ 11.78%) groups. Compared to the MET group, the EMC group had a greater rate of decline (*p* = 0.051) in the high baseline IMAT% group. However, in the low baseline IMAT% group, both the EXE (*p* = 0.031), and EMC groups (*p* = 0.042) showed a significant rate of decline, and there was no significant difference in the rate of decline between the EXE and EMC groups (Fig. 5b, c).

### Correlations between multiparameter MRI and laboratory indicators

**Fig. 6:**
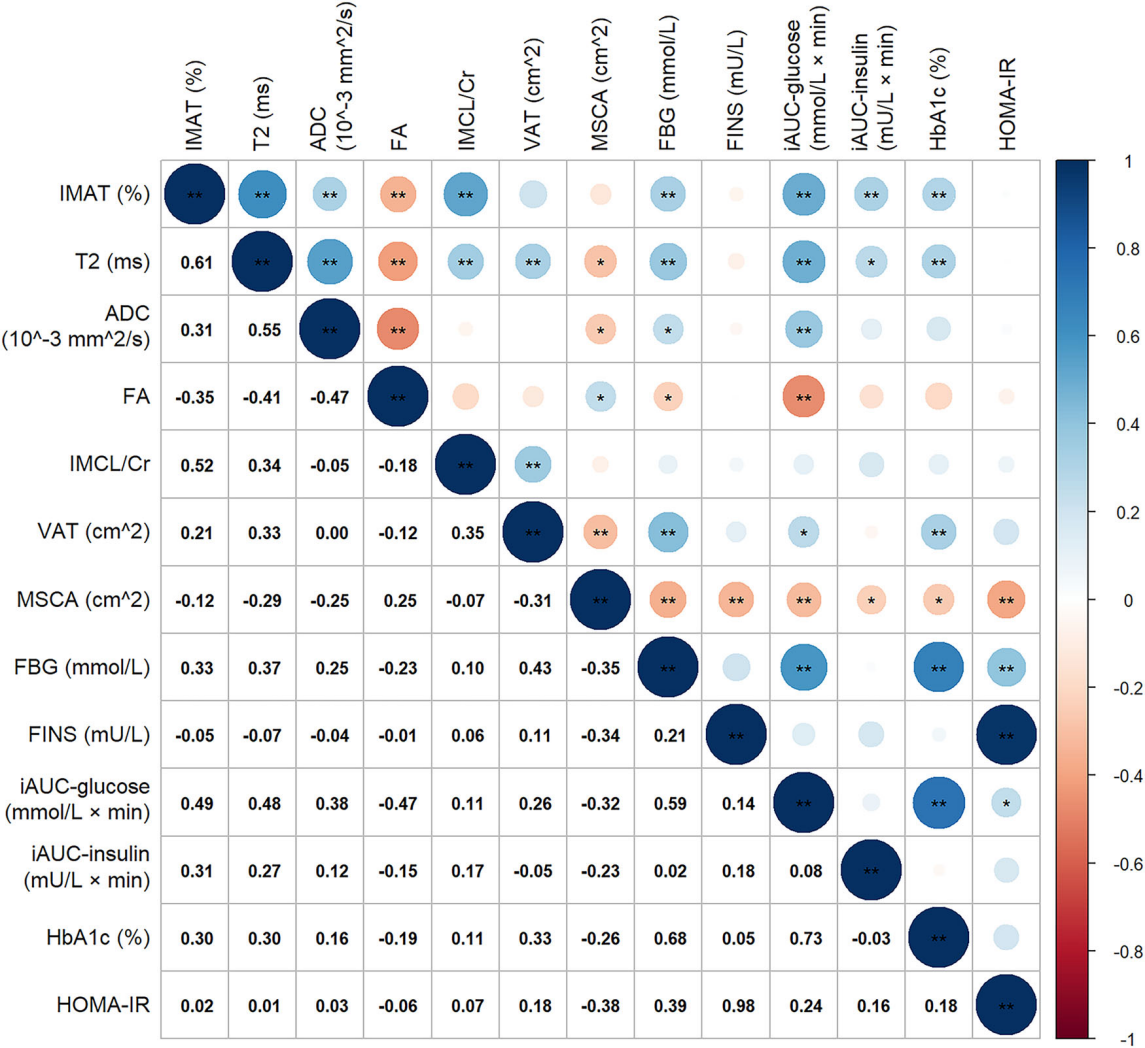
Bubble chart of post-treatment correlations between MRI metrics and blood biomarkers. ADC, Apparent diffusion coefficient; Cr, Creatinine; FA, Fractional anisotropy; FBG, Fasting blood glucose; FINS, Fasting blood insulin; HbA1c, Hemoglobin A1c; HOMA-IR, Homeostasis model assessment of insulin resistance; iAUC, Incremental area under the curve; IMAT, Intermuscular adipose tissue; IMCL, Intramyocellular lipid; MSCA, Muscle cross-sectional area; VAT, Visceral adipose tissue. * *p* < 0.05, ** *p* < 0.01^,^ statistically significant in correlation

After adjusting for BMI, sex, and age, correlations between MRI-derived muscle and adipose metrics and blood biomarkers were evaluated using data collected at week 12 (post-intervention) (Fig. 6). IMAT% and T2 values were significantly associated with FBG, HbA1c, iAUC-glucose, and iAUC-insulin levels. ADC and FA values were correlated with FBG and iAUC-glucose. VAT was associated with HbA1c and iAUC-glucose. MSCAs showed significant associations with FBG, FINS, HbA1c, iAUC-glucose, iAUC-insulin, and HOMA-IR levels.

## Discussion

In this study, we found that MIAE significantly improved SM metabolism, glucose control, and insulin sensitivity in individuals with prediabetes, as assessed by MRI parameters and standard metabolic markers. While metformin alone demonstrated limited efficacy, its combination with exercise led to greater reductions in VAT and IMAT%, as well as increased MSCAs, particularly among individuals with high baseline IMAT levels. Importantly, MRI parameters, including T2, ADC, FA, and MSCAs, showed strong correlations with key glycemic and insulin-related biomarkers, highlighting the utility of MRI as a noninvasive tool for evaluating the metabolic effects of lifestyle and pharmacologic interventions.

In the clinical treatment of prediabetes, combining MIAE and metformin often enhances glycemic control and insulin sensitivity [34]. However, recent studies on this approach have also shown mixed results regarding fasting and postprandial glycemic control, including cumulative, ineffective, or even opposite results [17, 35–37]. Based on our results, we observed that MIAE significantly improved IR in individuals with prediabetes, consistent with Færch et al, who reported that exercise primarily exerts its effects by enhancing insulin sensitivity [38]. In the combined treatments, after a 3-month combination study, Pilmark et al found that, in glucose-intolerant and healthy men, metformin had no significant effect on SM in terms of adapting to acute exercise or training [17]. Our study found that there was no further significant increase in steady-state blood glucose levels and insulin sensitivity in our combination-therapy group compared to the MIAE-alone group. Our findings agree with those of Ortega et al [39], who reported that metformin treatment did not attenuate the insulin-sensitizing effect of exercise, and that the potential additive impact of metformin in combination therapy was not significant. It is noteworthy that the PRE group, despite receiving nutritional counseling only, exhibited a mean BMI increase of +1.5 kg/m² and deterioration in glycemic markers over the 12-week period. Although randomized controlled trials often report neutral or modest weight changes in non-intervention arms, real-world observational data and cohort studies of prediabetes consistently demonstrate progressive metabolic worsening in the absence of active lifestyle intervention. Therefore, our findings also highlight the limited effectiveness of counseling alone.

Although SM is recognized as the principal target of interaction between metformin and aerobic exercise, the underlying mechanisms remain unclear. In this context, multiparameter MRI serves as a valuable and noninvasive tool for quantitatively assessing SM metabolic function, providing insight into treatment-induced changes beyond conventional blood-based biomarkers [40, 41].

Given that prediabetic SM often exhibits increased fat infiltration and tissue remodeling, both T2 and IMAT% values tend to rise with disease severity [42]. In our study, significant reductions in both T2 and IMAT% were observed in the EXE and EMC groups following intervention. T2 mapping was conducted without fat suppression; therefore, the measured T2 values likely reflect a combined influence of intramuscular lipid and water content. While T2 is sensitive to muscle edema and compositional alterations, it was used in this context as a surrogate marker of global muscle quality [43]. Our previous *in vivo* animal study using a prediabetic rat model demonstrated that SMs with elevated T2 values exhibited higher levels of intermuscular inflammatory markers and localized edema [28], supporting the relevance of T2 mapping in this setting. This suggests that the EXE and EMC groups both experienced meaningful improvements in muscle composition following intervention, potentially reflecting reduced fat infiltration and water content. Notably, no significant differences in T2 or IMAT% reduction were observed between the EXE and EMC groups, suggesting that the addition of metformin provided limited incremental benefit beyond exercise alone.

Our results also indicated that metformin preferentially increased the rate of IMAT decline in patients with higher baseline IMATs who exercised. As IMAT in SM is directly related to IR through the secretion of inflammatory cytokines [5, 44], it plays a vital role in SM-mediated IR and represents a potential therapeutic target for prediabetes. This implies that metformin may help patients with greater baseline IMAT% to benefit from MIAE. Thus, in the treatment of prediabetic patients with high baseline IMATs, the combination of MIAE and metformin may result in better IMAT reduction and improve the long-term prognoses of patients with prediabetes.

On the other hand, DTI provides a unique window into SM microarchitecture and function [45]. FA and ADC are sensitive markers of water diffusion and tissue organization, reflecting both intracellular-extracellular water mobility and capillary perfusion [46]. Prior studies have shown that these parameters correlate with muscle strength and energy metabolism [47]. As a metabolic disorder, prediabetes has been linked to early structural changes in SM, including endothelial and mitochondrial dysfunction and extracellular matrix (ECM) remodeling, which may drive insulin resistance and reduced lower-limb muscle strength [48]. Therefore, FA and ADC may serve as useful imaging biomarkers to detect early microstructural alterations in SM under metabolic stress. In our study, the EXE and EMC groups exhibited significantly lower ADC and higher FA values than the PRE and MET groups. Hence, our results suggest that MIAE may reduce edema and improve muscle fiber alignment, thereby increasing muscle anisotropy (higher FA values) while reducing the random diffusion of water molecules (lower ADC values).

Historically, the extent of IMCL degradation during exercise has been a subject of debate. In our study, we observed significant reductions in IMCL/Cr in both the EXE and EMC groups compared with the PRE and MET groups, consistent with recent meta-analytic findings showing robust IMCL depletion after aerobic exercise detected by ^1^H-MRS [49]. Regular aerobic exercise helps regulate IMCL turnover, maintaining dynamic balance and preventing the accumulation of lipotoxic intermediates [50]. Thus, combining therapy may help preserve lipid homeostasis in SM cells in prediabetes without impairing IMCL turnover.

Based on these results and our previous *in vivo* data [28], adding metformin during aerobic exercise did not substantially alter SM composition, nor did it confer additional benefits beyond those observed with exercise alone. This phenomenon may occur because metformin has a short accumulation and retention time in SM, adipocytes, and mitochondria [16]. Indeed, other researchers have reported no adverse effects on mitochondria at therapeutically relevant metformin concentrations (100 μM) *in vitro* [51, 52]. Thus, our data imply that metformin does not negatively influence overall SM metabolism.

Our study has several limitations. A major limitation is the relatively small sample size, which may constrain the external validity and generalizability of our findings. Future studies with larger cohorts are warranted to confirm and extend our results. In addition, we did not perform SM biopsies because we aimed to avoid invasive procedures and minimize potential discomfort for participants with pre-diabetes. Moreover, we used HOMA-IR, a simpler and less invasive method, instead of the euglycemic-hyperinsulinemic clamp technique, which may not fully capture the complete range of metabolic responses to metformin and exercise. Finally, we did not implement detailed longitudinal quantification of alcohol use beyond baseline screening and pre‑assessment abstinence instructions, which may allow minor unmeasured variation in low-level intake.

In conclusion, combination therapy is recommended for prediabetes management, as exercise improves insulin sensitivity and glucose homeostasis, while metformin, when administered under medical supervision, may additionally contribute to reductions in IMAT, particularly in individuals with high baseline levels and elevated metabolic risk. Multiparameter MRI offered quantitative insights, with key parameters correlating with glucose and insulin disturbances, highlighting its utility as a noninvasive tool for monitoring metabolic changes and treatment response.

## Supplementary information

**Additional file 1:** **Table S1.** Magnetic resonance sequence acquisition parameters. **Table S2.** Intra- and inter-reader ICC and 95% confidence interval.

## Data Availability

The dataset supporting the findings of this study, “Multiparameter MRI Assessment of Metformin and Exercise Effects on Skeletal Muscle in Prediabetes: A Randomized Controlled Trial”, is available on Mendeley Data at 10.17632/9zf89cpnh3.6. All MRI and laboratory data have been fully de-identified to remove personal information, ensuring patient privacy and ethical compliance.

## Abbreviations

ADC: Apparent diffusion coefficient
BMI: Body mass index
DTI: Diffusion tensor imaging
EMC: Combined metformin and exercise group
EXE: Exercise-only group
FA: Fractional anisotropy
FBG: Fasting blood glucose
FINS: Fasting blood insulin
HbA1c: Hemoglobin A1c
HOMA-IR: Homeostasis model assessment of insulin resistance
iAUC: Incremental area under the curve
ICC: Intraclass correlation coefficient
IMAT: Intermuscular adipose tissue
IMCL: Intramyocellular lipids
MET: Metformin group
MIAE: Moderate-intensity aerobic exercise
MRI: Magnetic resonance imaging
MRS: Magnetic resonance spectroscopy
MSCA: Muscle cross-sectional area
PRE: Prediabetes group
ROI: Region of interest
SM: Skeletal muscle;
VAT: visceral adipose tissue
