# Supplementary material for: Multiparameter MRI assessment of metformin and exercise effects on skeletal muscle in prediabetes: a randomized controlled trial
Source: Eur Radiol Exp. 2025 Dec 22;9:123. doi: 10.1186/s41747-025-00658-y (PMC12722603; doi:10.1186/s41747-025-00658-y)
Supplement: Supplementary file 1 — Additional file 1: Table S1. Magnetic resonance sequence acquisition parameters. Table S2. Intra- and inter-reader ICC and 95% confidence interval. [file 41747_2025_658_MOESM1_ESM.pdf]

Multiparameter MRI assessment of metformin and exercise effects on skeletal muscle in prediabetes: a randomized controlled trial

ELECTRONIC SUPPLEMENTARY MATERIAL

Table S1. Magnetic resonance sequence acquisition parameters

Scanning parameters	T1W1	T2W1	DTI	T2 mapping	6-echo Dixon#	^{1}H-MRS
Orientation	Sagittal/Coronal/Axial	Sagittal/Coronal/Axial	Axial	Axial	Axial	Axial
TR (ms)	260	5384	3357	2010	9.1	2000
TE (ms)	15	100	84	40/60/80/100	1.3/2.6/3.9/5.2/6.5/7.8	50
FOV (mm)*	400×400×150	400×400×150	410×410×155	414×420×160	420×420×180	N/A
NEX	3	2	2	2	2	128
Slice thickness (mm)	5	5	4	5	6	N/A
In-plane resolution (mm)	1.25×1.25	1.25×1.25	1.5×1.5	1.3×1.3	0.82 ×0.82	N/A
Matrix size (Acq.)	320×320	320×320	272×272	320×320	512×512	N/A
Voxel size (mm)	1.25×1.25×5.0	1.25×1.25×5.0	1.5×1.5×4	1.3×1.3×5.0	0.82×0.82×1.0	30×30×30
b-values (s/mm²)	N/A	N/A	0, 500	N/A	N/A	N/A
Diffusion directions	N/A	N/A	15 + 1 b0	N/A	N/A	N/A
SNR (ROI / voxel)	40 (voxel)	45 (voxel)	103 (ROI, b0)	35 (voxel)	50 (voxel)	N/A
MRS fitting method	N/A	N/A	N/A	N/A	N/A	LCModel
Scanning time (min:s)	2:09	2:10	3:13	2:13	00:09	5:37

Acq, acquisition matrix; DTI, diffusion tensor imaging; FOV, field of view; MRS, magnetic resonance spectroscopy; NSA, number of excitations; ROI, region of interest; SNR, signal-to-noise ratio; TE, echo time; TR, repetition time. N/A indicates that the parameter is not applicable or was not acquired for the given sequence.

*FOV values list in-plane dimensions followed by craniocaudal coverage (third value). #The 6-echo Dixon sequence used six gradient echoes (TE1–TE6 as listed) for multi-echo water–fat separation; acquisition matrix 420 × 420 reconstructed to 512 × 512 yielding 0.82 mm in-plane resolution.

Table S2. Intra- and inter-reader ICC and 95% confidence interval

Parameters	Intra-reader		Inter-reader	
	ICC	95% CI	ICC	95% CI
T2 (ms)				
Week 0	0.83	0.74–0.90	0.92	0.87–0.96
Week 12	0.88	0.81–0.93	0.85	0.76–0.91
ADC (10^{-3} mm^2/s)				
Week 0	0.90	0.85–0.95	0.93	0.89–0.96
Week 12	0.82	0.71–0.90	0.83	0.73–0.90
FA				
Week 0	0.91	0.84–0.95	0.93	0.90–0.96
Week 12	0.81	0.70–0.89	0.94	0.89–0.96
VAT (cm^2)				
Week 0	0.96	0.94–0.98	0.93	0.89–0.96
Week 12	0.94	0.91–0.96	0.89	0.83–0.94
IMAT%				
Week 0	0.97	0.96–0.98	0.96	0.93–0.97
Week 12	0.81	0.70–0.88	0.84	0.74–0.90
MSCA (cm^2)				
Week 0	0.98	0.62–0.98	0.96	0.60–0.99
Week 12	0.96	0.60–0.97	0.92	0.70–0.99

ADC, apparent diffusion coefficient; CI, confidence interval; FA, fractional anisotropy; IMAT, intermuscular adipose tissue; MSCA, muscle cross-sectional area; VAT, visceral adipose tissue.
